# 

**Published:** 1890-03

**Authors:** 


### CONTENTS—MARCH, 1890.—VOL. 5, NO. 9.

ORIGINAL COMMUNICATIONS— Page

Life, (concluded). W. A. Morris, M. D. . . . . . . . . . . 321

Diagnosis in Diseases. B. F. Brittain, M. D. . . . . . . . 329

A Case of Triplets. E. D. Chase, M. D. . . . . . . . . . . 330

CORRESPONDENCE—

A Week of Practice in Mexico—The Doctors' Paradise (?)
E. G. Cochran, M. D. . . . . . . . . . . . . . . . . . 332

Quackery Rampant. R. E. Moss, M. D. . . . . . . . . . . 334

Further Success with Nitre as a Remedy in Chill and Fever.
J. D. Hunter, M. D. . . . . . . . . . . . . . . . . . 336

SOCIETY NOTES—

Johnson County Medical Society . . . . . . . . . . . . 338

Concerning the Tenth International Medical Congress . . 340

Cherokee Medical Association . . . . . . . . . . . . . . 34[illegible]

The Terrell Medical Society. . . . . . . . . . . . . . . 344

Texas State Medical Association—Arrangements for the
Coming Meeting, April 22 . . . . . . . . . . . . . . 345

American Medical Association. . . . . . . . . . . . . . 346

Texas Sanitary Association . . . . . . . . . . . . . . . 348

EDITORIAL—

Raising the Standard of the Medical Education—the Way
to Do It . . . . . . . . . . . . . . . . . . . . . . . 349

MEDICAL NEWS AND MISCELLANY—

Personals, Removals, etc. . . . . . . . . . . . . . . . 352

Some Recent Additions to the Materia Medica. . . . . . 354

BOOK NOTICES—

Notices and Reviews . . . . . . . . . . . . . . . . . . 357

PUBLISHER'S NOTES—

Of Interest to Practitioners and Others . . . . . . . . . 359

